# P-2108. Clinical Outcome differences in Kidney Transplant Patients Receiving Letermovir Versus Valganciclovir for CMV Prophylaxis

**DOI:** 10.1093/ofid/ofaf695.2272

**Published:** 2026-01-11

**Authors:** YoungYoon Ham, Ali Olyaei, Ismael Aguirre

**Affiliations:** Oregon Health & Science University, Portland, Oregon; Oregon Health and Science University (OHSU), Portland, Oregon; Oregon Health and Science University (OHSU), Portland, Oregon

## Abstract

**Background:**

CMV is a common herpesvirus that remains latent after initial infection. Adult seroprevalence ranges from 45-100% globally and is higher in transplant recipients (∼62%). CMV threatens immunocompromised patients, especially solid organ transplant recipients. Prophylaxis with valganciclovir or ganciclovir is standard but causes hematologic toxicity. Letermovir, FDA-approved in 2023 for high-risk kidney recipients, showed similar efficacy with fewer side effects.

**Figure:**
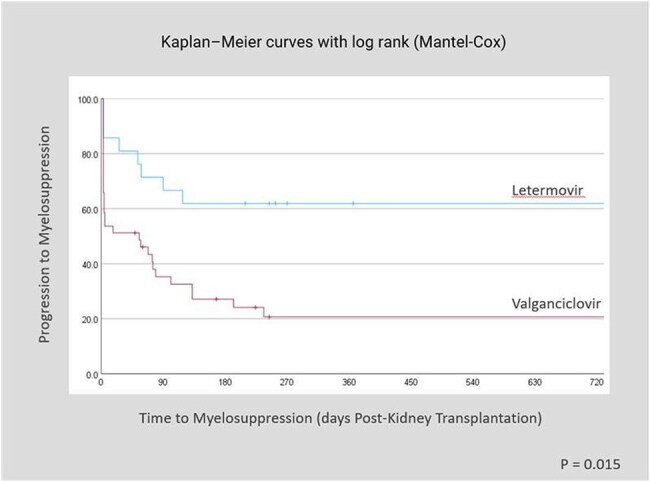
Time to Myelosuppression (days Post-Kidney Transplant)

**Figure:**
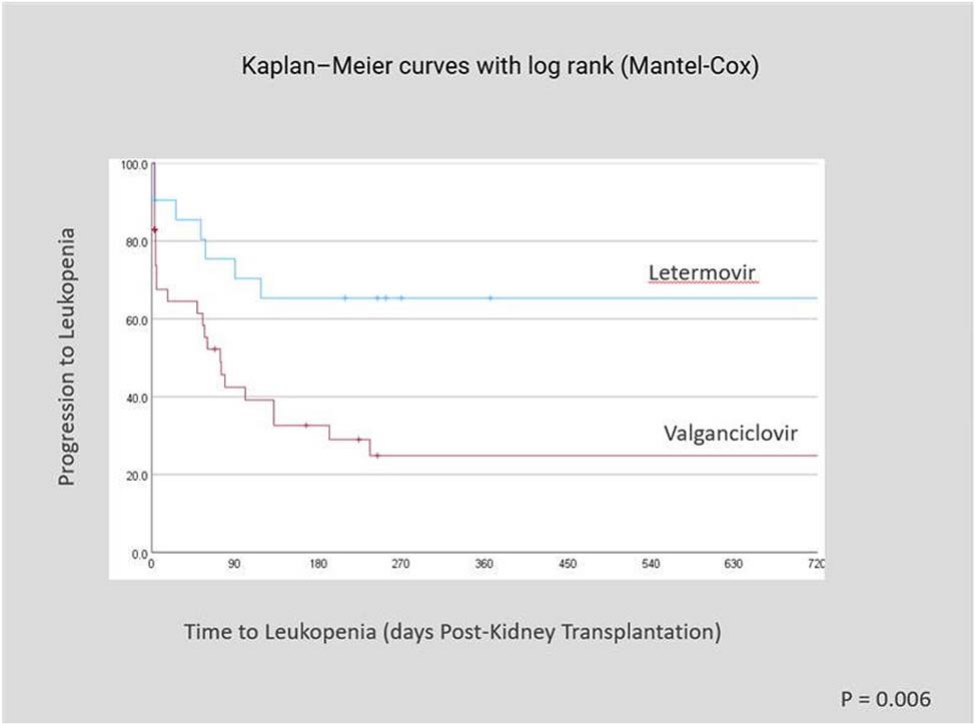
Time to Leukopenia (days Post-Kidney Transplant)

**Methods:**

This single-center, retrospective chart review at Oregon Health & Science University evaluated adults who underwent kidney transplantation and were prescribed letermovir or valganciclovir for CMV prevention between June 20, 2022, and December 1, 2024. Exclusions included pediatric, pregnant, or VA patients, those who died within six months post-transplant, or did not complete prophylaxis. Data were collected from EPIC and the UNOS database, including demographics, clinical outcomes, and adverse events. The primary outcome was CMV viremia, secondary outcomes included safety and tolerance.

**Results:**

A total of 62 pt, 21 in letermovir group and 41 in valganciclovir group.

Breakthrough CMV viremia within 6 months of kidney transplantation occurred in 1 of 21 patients (4.8%) receiving letermovir prophylaxis compared to 6 of 41 patients (14.6%) receiving valganciclovir. No patients were admitted for CMV related disease or complications. Leukopenia was observed in 24% of patients in the letermovir group versus 57% in the valganciclovir group (p = 0.006), while neutropenia occurred in 39% of letermovir-treated patients compared to 61% of those treated with valganciclovir (p = 0.015).

**Conclusion:**

Findings from this study are consistent with prior clinical trials demonstrating the non-inferiority of letermovir for CMV prophylaxis in kidney transplant recipients. Letermovir showed reduced myelosuppression including leukopenia and neutropenia. In addition, our results seemed to suggest lower rates of breakthrough CMV viremia with letermovir, but no clinically significant CMV disease was observed in either group.

**Disclosures:**

All Authors: No reported disclosures

